# Alternative Splicing Targeting the hTAF4-TAFH Domain of TAF4 Represses Proliferation and Accelerates Chondrogenic Differentiation of Human Mesenchymal Stem Cells

**DOI:** 10.1371/journal.pone.0074799

**Published:** 2013-10-02

**Authors:** Jekaterina Kazantseva, Anri Kivil, Kairit Tints, Anna Kazantseva, Toomas Neuman, Kaia Palm

**Affiliations:** 1 Protobios LLC, Tallinn, Estonia; 2 The Department of Gene Technology, Tallinn University of Technology, Tallinn, Estonia; The Chinese University of Hong Kong, China

## Abstract

Transcription factor IID (TFIID) activity can be regulated by cellular signals to specifically alter transcription of particular subsets of genes. Alternative splicing of TFIID subunits is often the result of external stimulation of upstream signaling pathways. We studied tissue distribution and cellular expression of different splice variants of TFIID subunit *TAF4* mRNA and biochemical properties of its isoforms in human mesenchymal stem cells (hMSCs) to reveal the role of different isoforms of TAF4 in the regulation of proliferation and differentiation. Expression of *TAF4* transcripts with exons VI or VII deleted, which results in a structurally modified hTAF4-TAFH domain, increases during early differentiation of hMSCs into osteoblasts, adipocytes and chondrocytes. Functional analysis data reveals that TAF4 isoforms with the deleted hTAF4-TAFH domain repress proliferation of hMSCs and preferentially promote chondrogenic differentiation at the expense of other developmental pathways. This study also provides initial data showing possible cross-talks between TAF4 and TP53 activity and switching between canonical and non-canonical WNT signaling in the processes of proliferation and differentiation of hMSCs. We propose that TAF4 isoforms generated by the alternative splicing participate in the conversion of the cellular transcriptional programs from the maintenance of stem cell state to differentiation, particularly differentiation along the chondrogenic pathway.

## Introduction

Transcription initiation by RNA polymerase II requires assembly of general transcription factors (GTFs) to form a functional preinitiation complex (PIC). Recent data shows that the composition of the general transcriptional machinery is not static but spatio-temporally regulated during development of different tissues [1]. Genetic, functional and biochemical studies using different cell types and model organisms have revealed the existence of the alternative initiation complexes [2].

Transcription factor complex TFIID is one of the main components of the general transcriptional machinery. It consists of TATA binding protein (TBP) and up to 14 TBP-associated factors (TAFs) [3]. TFIID is essential for promoter recognition and interactions with transactivators [4]. Most recently, it was established that the human TFIID core complex contains two copies each of TAF4, TAF5, TAF6, TAF9 and TAF12 [5]. TBP and TAFs are highly regulated [6], [7], whereas modified forms of TFIID selectively act on specific transcriptional networks. For example, TAF4b in cooperation with c-Jun drives tissue-specific programs of gene expression [8], whereas TAF10 is essential for the expression of a subset of genes required for cell cycle progression [9]. The loss of a specific TAF function always affects a defined subset of genes [10] indicating that each TAF has a different and distinct role in transcription of certain but not all genes.

TAF4 plays a critical role in maintaining TFIID complex structural stability [11]. Metazoan TAF4 protein has conserved the N-terminal glutamine-rich domain followed by the co-activator TAF4-homology ETO-TAFH/CRI/NHR1 (TAFH) domain in the central part of the protein and C-terminal histone-like (CRII) domain [12]. The four glutamine-rich domains of human TAF4 have been shown to mediate interactions with activators CREB [13] and Sp1 [14]–[16]. The interactions of TAFH domain with N-CoR and its repressive activity on transcription through its interaction with E-proteins have been reported [17]. However, the human TAF4 TAFH domain (hTAF4-TAFH) represents a five-helix structure that is characteristic to vertebrates only and therefore has a distinct although related interaction specificity from that of the other TAFH domains [18]. The role of HDACs, methyltransferases, and the PBX family of transcription factors as interaction partners of hTAF4-TAFH has been postulated [18]. The importance of TAFH domain in WNT signaling in *Drosophila* has been established [19], suggesting the role of hTAF4-TAFH in development, cell fate determination and differentiation.

Structural and functional diversity of cell-specific GTFs and complex molecular mechanisms regulating their activity during development and differentiation are well described. Data on the impact of individual TFIID subunits on cell differentiation are controversial [20]–[22]. In *C. elegans*, TAF4 in cooperation with zinc finger proteins OMA-1/2 drives global repression of transcription and regulates oogenesis [23]. Undifferentiated embryonic stem cells express only a subset of canonical TAFs, lacking TAF4 [24]. Regulated TAF4 degradation has been reported to be essential for male germ-cell differentiation [25]. TAF4b, a TAF4 paralog, is required for oocyte development [26] and participates in the proper maintenance of spermatogenesis in the mammalian testis [27]. No data on TAF4 developmental expression patterns and the functional effects of directed silencing and deregulation of the hTFA4-TAFH domain have been available up to now.

During developmental signaling, alternative splicing resulting in distinct protein isoforms with specific biochemical properties are a prevalent mechanism in modulating the function of many transcription factors. Alternatively spliced mRNAs are identified for all TAF subunits [28]. Nevertheless, only a few studies have addressed the role of TAF protein isoforms in a cell and tissue specific context. In humans, TAF6δ isoform, in opposed to major TAF6α isoform, triggers apoptosis [7]. In *Drosophila*, two isoforms, TAF1-1 and TAF1-2, differ in their DNA binding activities and contribution to gene-specific transcription [29]. Currently, five alternatively spliced murine *Taf4* mRNAs have been described [30]. These splice variants encode protein isoforms that differ in the structure of their TAFH and CRII domains and affinity for different nuclear receptors. Some are expressed in a cell-type specific manner and exhibit dominant negative effects on nuclear receptor-mediated TAF4 transcription.

Here we describe functional consequences of alternative splicing of TAF4 affecting the integrity of the hTAF4-TAFH domain on human mesenchymal stem cell proliferation and differentiation. Our data suggests that alternative splicing of *TAF4* is one of the key processes influencing stem cell differentiation and reprogramming.

## Materials and Methods

### Ethics Statement

Experiments using human material were approved by the Ethics Committee of the National Institute for Health Development, Tallinn, Estonia (Approval No 2234 from Dec 09, 2010) and written informed consent was obtained from all participants.

### Cell culture

Human MSCs were obtained from freshly isolated subcutaneous adipose tissue as previously reported [31] and published by Kauts et al. [32]. Description of the donors and the use of human cells in different studies are provided in Table S1. The cells were expanded in a medium consisting of low glucose Dulbecco's modified Eagle's medium with glutamine (DMEM-LG) (PAA Laboratories, Austria) supplemented with 1% penicillin/streptomycin (PAA Laboratories) and 10% fetal bovine serum (FBS) (PAA Laboratories) in a humidified atmosphere at 37°C and 5% CO_2_. The first plating of the cells after the isolation was designated as passage 0 (P0), and each splitting of confluent cells was considered as the next passage. The cells from passages between P2 and P5 were used in the functional assays in the present study.

### siRNA transfection

Small interfering RNAs (siRNAs) targeting exons V and VI of *TAF4* were designed as 5′-GGUUAUACCGAGAACUUAA-dTdT-3′ and 5′- CAGCUAAUGUGAAAGAGCU-dTdT-3′ respectively. *Silencer*® Select *TAF4* siRNAs and Negative Control #2 synthetic scrambled siRNAs were purchased from Ambion, Invitrogen (UK). hMSCs were trypsinized 24 h before transfection and treated with 50 nM of each siRNA using Lipofectamin RNAiMAX reagent (Invitrogen, UK) according to the manufacturer's protocol. Normal growth medium was added 24 h after transfection and changed every three days. All experiments were done using siRNAs targeting exons V and VI of *TAF4*, whereas results generated using siRNAs targeting exon VI of *TAF4* were used for data presentation.

### Differentiation Procedures

Approximately 90% confluent hMSCs at passages P2 to P5 were transfected with *TAF4* or control siRNAs and 24 h later treated with adipogenic, chondrogenic or osteogenic type of differentiation media for up to 7 days. Adipogenic differentiation medium consisted of DMEM/F-12 (Gibco, Invitrogen, UK) supplemented with 5% heat-inactivated FBS, 10 µg/ml insulin (Sigma, USA), 0.5 mM IBMX (Sigma, USA), 0.1 mM indomethacin (Sigma, USA) and 1 μM dexamethasone (Sigma, USA). Osteogenic differentiation medium consisted of DMEM-F12 supplemented with 5% heat-inactivated FBS, 10 nM BMP6 (PeproTech, USA), 10 nM dexamethasone, 10 mM and β-glycerolphosphate (Sigma, USA). Chondrogenic differentiation medium consisted of DMEM-High Glucose (PAA Laboratories) supplemented with 10 nM TGF-β1 (PeproTech, USA), 0,1 µM dexamethasone, 1 mM ascorbic acid-2-phosphate (Sigma, USA), 1 mM sodium pyruvate (Gibco, Invitrogen, UK) and 1% insulin-transferrin-selenium-X (Gibco, Invitrogen, UK). Lipid-rich vacuoles were visualized using Oil-Red-O (Sigma, USA) staining performed as described [33]. Osteogenic differentiation was assessed using alkaline phosphatase substrate NBT/BCIP (Pierce Biotechnology Rockford, IL, USA) as described [34]. Chondrogenic differentiation was assessed by immunofluorescence staining.

### Immunofluorescence

hMSCs were grown on 22-mm^2^ glass slides to about 70% confluency, treated with control or *TAF4* siRNAs and induced with differentiation supporting media for chondrogenic differentiation for 5 days. Cells were washed once with 1xPBS, fixed using 4% paraformaldehyde (Scharlau, Germany) in 1xPBS for 20 min at RT, washed 3 times with 1xPBS and blocked in 1xTBS containing 0.05% Tween20 (TBS-T) and 2% of bovine serum albumin (BSA) for 2 h at RT. Primary antibodies against COL2A1 (Millipore, MAB1330, 1∶100) and SOX9 (Millipore, AB5535, 1∶500) were diluted in 1xTBS containing 0.01% Tween20 and 0.2% BSA. Cells were incubated with primary antibodies for 2 h at RT, washed three times with TBS-T and incubated with anti-rabbit Alexa Flour 546 or anti-mouse Alexa Flour 488 secondary antibodies (Molecular Probes, Invitrogen, UK) for 1 h at RT in the dark. Cells were washed three times with TBS-T and mounted using ProLong Gold antifade reagent (Invitrogen, UK). Images were obtained using a Nikon Eclipse 80i fluorescence microscope (Nikon Instruments Inc., USA).

### RNA isolation, RT-PCR and Real-Time PCR

A normal human tissue RNA panel was purchased from BioChain Institute Inc. (CA, USA). Total RNA was purified using Trizol reagent (Invitrogen, UK) following the manufacturer's recommendations. The RNA concentration was determined using a NanoDrop ND-1000 instrument (Thermo Scientific, USA). cDNA was synthesized from DNase-treated (Ambion, Invitrogen, UK) RNA with Superscript III (Invitrogen, UK) and mixture of oligo dT and random hexamers, according to the manufacturer's recommendations. RT-PCR was carried out using HOT FIREpol® Master Mix (Solis Biodyne, Estonia). Real-time PCR (qRT-PCR) was performed in triplicate using Platinum® SYBR® Green qPCR SuperMix-UDG (Invitrogen, UK) and the LightCycler® 480 Real-Time PCR System (Roche Applied Science). The fold of change was calculated relative to the control siRNAs after normalization to *GAPDH* expression. Primer sequences are listed in Table S2.

### Protein extraction and immunoblot

*TAF4* or control siRNA-treated cells were collected by trypsin-EDTA (PAA Laboratories) and washed once with ice-cold 1xPBS. Cell fractionation was carried out according to modified Dignam protocol [35] using 0.2% Nonidet P-40 in the lysis buffer as described by Kazantseva et al. [36]. Total protein concentration of nuclear lysates or whole cell extracts was measured using a BCA Protein Assay kit (Pierce Biotechnology Rockford, IL, USA). Equal amounts of total protein in a nuclear lysate or a whole cell extract were separated on 10% polyacrylamide gel and blotted to PVDF membrane (GE Healthcare). The membrane was treated as described [37] using a non-blocking technique. The following antibodies were used: TAF4 (BD Biosciences, 612054), CDKN1A (Santa Cruz, sc-756), TP53^Ser15^ (Cell Signaling, 9284), ADIPOQ (Chemicon, MAB3604), PPARG2 (Chemicon, MAB3872), RUNX2 (Abcam, ab76956), OPN (Santa Cruz, sc-10591), COL21A (Millipore, MAB1330), MMP13 (Biomol, SA-371), SOX9 (Millipore, AB5535), ß-catenin (Santa Cruz, sc-7963) and GAPDH (Sigma, G8795) antibodies. Secondary HRP-conjugated antibodies were purchased from Abcam (UK). Proteins were visualized using SuperSignal West Pico Chemiluminescent Substrate (Pierce Biotechnology Rockford, IL, USA).

### WST-1 Cell Proliferation Assay

hMSCs were grown in 96-well flat bottom tissue culture plates to 80% confluency and treated with *TAF4* or control siRNA. WST-1 reagent (Roche Applied Science) was added following the manufacturer's instructions and plates were returned to 37°C for 2 h. Dye conversion was measured using SPECTRAmax 340 PC Microplate Reader (Molecular Devises LLC, USA) and the data were analyzed using Softmax Pro 3.12 software. Cell viability was evaluated every 24 h, up to 96 h post-treatment.

### Cell cycle analysis

hMSCs after siRNA treatments were harvested by trypsinization, washed with 1xPBS and counted using a NucleoCounter NC-100 (Chemometec, Denmark). Approximately 4×10^5^ cells were resuspended in 1 ml of 1xPBS containing 2% FBS and fixed with ice-cold ethanol (70% v/v) overnight at -20°C. Cell pellets were exposed to 100 μg/ml of RNaseA (Fermentas, Thermo Scientific) and 40 μg/ml of Propidium Iodide (AppliChem GmbH) in 1xPBS for 30 min at RT in the dark. Cell cycle distribution was assessed using Accuri C6 flow cytometer (BD Biosciences). For each sample, 10 000 individual events were collected.

### Senescence-associated ß-galactosidase assay

Senescence-associated ß-galactosidase (SA- ß-gal) activity was examined as previously described [38]. Cells treated with 1 µM 4-NQO (Sigma) were used as a positive control. SA- ß-gal activity was analyzed 48 h after siRNA treatment.

## Results

### Tissue- specific alternative splicing of human *TAF4* targeting TAFH domain

**Figure 1:**
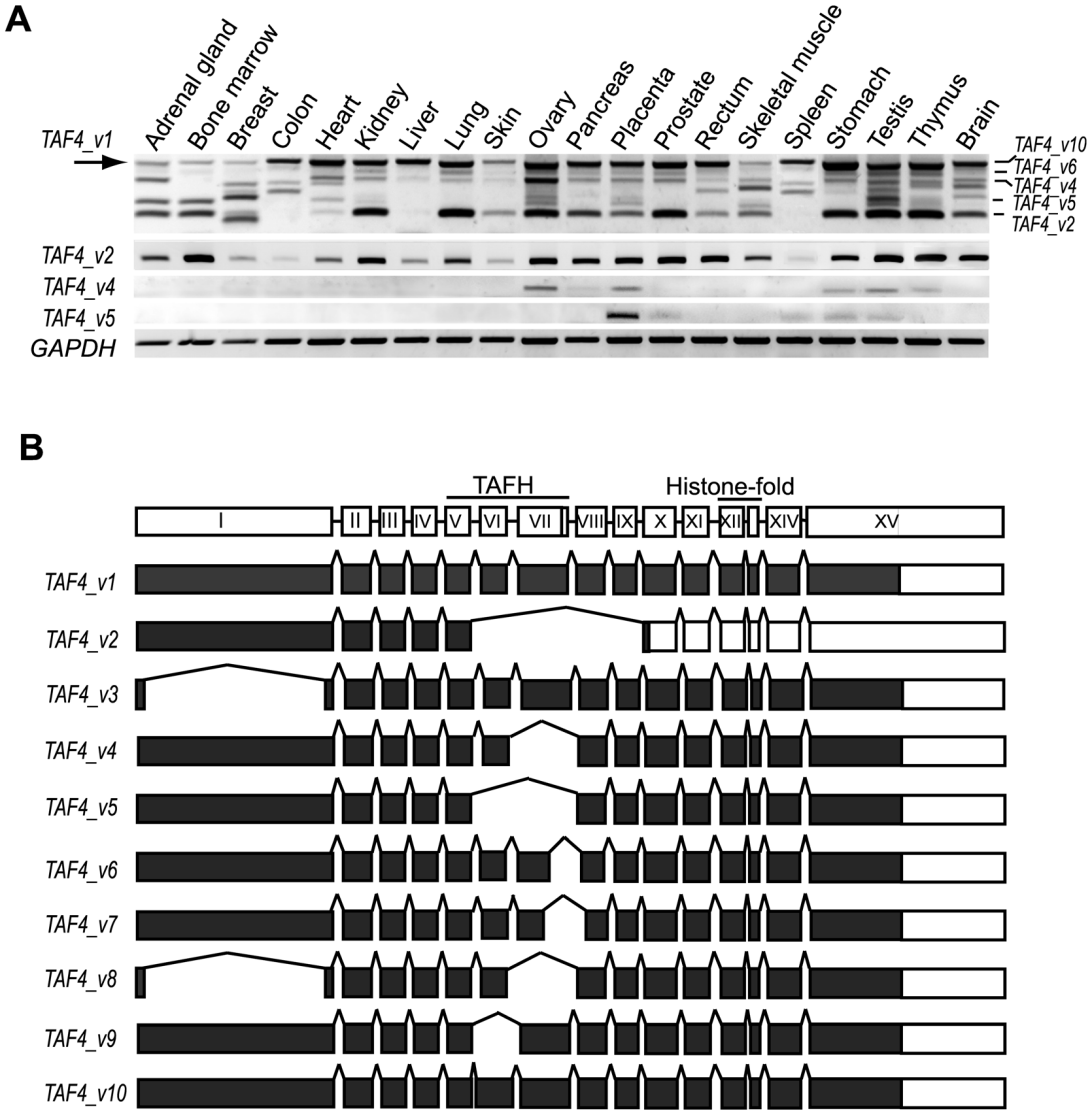
Expression analysis of *TAF4* alternative splice variants in human tissues. (**A**) RT-PCR analysis of human tissues using *TAF4* transcript-specific primers. Primers for the full-length *TAF4_v1* amplify all splice variants, whereas ASV-specific primers generate predominantly one PCR product. The numbers of PCR cycles and exposure times of the images for each set of primers vary and cannot be directly compared. The arrow indicates the canonical *TAF4_v1* ASV. (**B**) Schematic representation of the human *TAF4* gene structure and its alternative splice variants drawn in scale. The regions encoding the respective domains are indicated above the gene structure. Filled boxes represent the coding regions of the ASVs.

To identify tissue-specific expression patterns of human *TAF4* mRNA splice variants, RT-PCR method with subsequent sequencing analyses of PCR fragments was used. Sequence analysis of the RT-PCR products revealed a variety of tissue-specific splice variants preserving the reading frame **(** **Figure** **1** **)**. However, a significant number of *TAF4* alternatively spliced mRNAs contained a premature termination codon, indicating that these splice variants are subject to nonsense-mediated RNA decay (data not shown). Tissue-restricted splicing patterns of *TAF4* containing in-frame splicing events encoding protein isoforms are shown in **Fig.** **1A**. Schematic presentation of ten different TAF4 isoforms encoded by different splice variants is shown in **Figure** **1B**. Interestingly, alternative splicing frequently targets exons VI and VII encoding the hTAF4-TAFH domain **(** **Fig.** **1B** **)**. As shown in **Figure** **1**, the structure of the hTAF4-TAFH domain is apparently different in all isoforms except *TAF4_v1* and *TAF4_v3*, as evidenced by sequence analysis of identified *TAF4* mRNA alternative splice variants (ASVs) containing exonal deletions (*TAF4_v2, TAF4_v4-9*) or extensions (*TAF4_v10*) with ORF preservation. Transcripts *TAF4_v3* and *TAF4_v8* differ also in their N-terminal part.

In many tissues examined, certain alternatively spliced *TAF4* mRNAs were expressed approximately at the same levels. Two *TAF4* ASVs, *TAF4_v1* and *TAF4_v2*, were the most abundant and the most broadly expressed splice variants detected in all tissues analyzed **(** **Fig.** **1A** **)**. *TAF4_v1* corresponds to the longest transcript (GenBank *NM_003185.3*). Alternatively spliced *TAF4* mRNAs with altered hTAF4-TAFH domains exhibit distinct patterns of tissue-specific expression. Alternatively spliced *TAF4_v2* mRNAs containing simultaneously a deletion of exons VI – IX and an in-frame stop codon in the exon X encode TAF4 protein isoforms with the entire hTAF4-TAFH domain deleted. The ratio and levels of *TAF4_v1* and *TAF4_v2* expression varied across tissues with *TAF4_v2* dominating in bone marrow, kidney, ovary, placenta, prostate, testis and thymus and barely detectable in colon, skin and spleen. Other alternative mRNA splice variants of *TAF4* were highly tissue-specific. *TAF4_v4* mRNAs lacking exon VII that encodes a part of the hTAF4-TAFH domain were identified only in ovary, placenta, stomach, testis and thymus **(** **Fig.** **1A** **)**. Expression of *TAF4_v5* mRNAs with deleted exon VI encoding the major part of the hTAF4-TAFH domain overlapped with expression of *TAF4_v4* in all tissues except for ovary **(** **Fig.** **1A** **)**.

### Differentiation of mesenchymal stem cells to adipocytes, osteoblasts and chondrocytes is associated with expression of *TAF4* transcripts with the deleted hTAF4-TAFH domain

**Figure 2:**
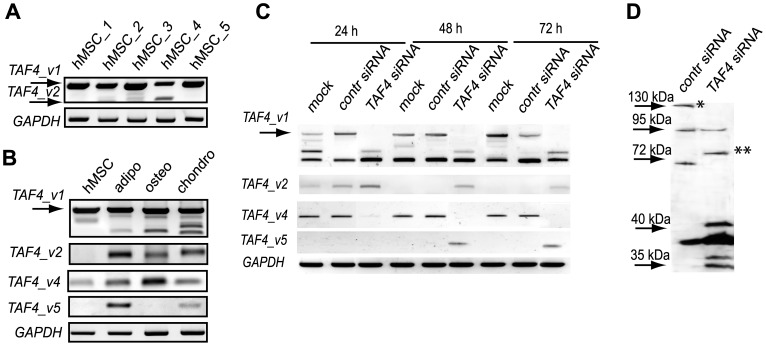
Expression of TAF4 splice variants and isoforms in human MSCs differentiated into adipocytes, osteoblasts or chondrocytes and treated with *TAF4* RNAi. (**A**) RT-PCR analysis of different individual hMSCs clones (hMSCs 1–5) using *TAF4_v1a* (full length) specific primers (see Table S2). Expression of *GAPDH* mRNA is shown at the bottom. (**B**) Expression of *TAF4* ASVs encoding proteins with compromised hTAF4-TAFH domain is dominant in differentiated hMSCs. RT-PCR analysis using *TAF4* ASV-specific primers was performed 7 days after induction of differentiation of hMSCs towards adipo-, osteo- and chondrogenic lineages. *GAPDH* mRNA expression was used for the normalization. (**C**) Expression of *TAF4* ASVs following treatment of human MSCs with control and hTAF4-TAFH-domain targeting *TAF4* siRNAs. Cells were treated with *TAF4* or control siRNAs at the indicated time points and RT-PCR analysis performed using *TAF4* ASV-specific primers. Analysis of *GAPDH* mRNA expression was used for normalization. (**D**) siRNA-mediated silencing targeting *TAF4* exons V or VI induces changes in the expression of TAF4 protein isoforms as detected at 48 h post-treatment using Western blot analysis. The asterisk indicates the canonical form of TAF4 protein with the molecular weight of 135 kDa, two asterisks indicate TAF4_v2 isoform with a calculated molecular weight of about 73 kDa.

Analysis of expression of *TAF4* splice variants revealed dominant expression of *TAF4_v1* in proliferating hMSCs **(** **Fig.** **2A** **)**. Splice variant *TAF4_v2*, which encodes a protein isoform with the entire hTAF4-TAFH removed, showed different expression in hMSCs isolated from different individuals. It was detected at a low level only in a few hMSC isolates, indicating that *TAF4_v2* is a rare transcript **(** **Fig.** **2A** **)**. Alternative splice variants of *TAF4* encoding for proteins with a structurally altered hTAF4-TAFH domain become more abundant in the course of differentiation of hMSCs into adipocytes, osteoblasts and chondrocytes **(** **Fig.** **2B** **)**. Expression of *TAF4_v2* ASV was observed in all differentiated hMSCs together with *TAF4_v1* and other splice variants **(** **Fig.** **2B** **)**. The data clearly shows that hMSC differentiation along adipo-, osteo- and chondrogenic lineages was accompanied by changes in the expression of *TAF4* mRNA splice variants with a structurally altered hTAF4-TAFH domain.

RNAi analysis was carried out using two structurally different siRNAs targeting exons V or VI in N-terminal half of the hTAF4-TAFH domain of *TAF4* to evaluate the role of the hTAF4-TAFH domain in differentiation of hMSCs (**Fig.** **2C**). We verified, using semi-quantitative RT-PCR and Western blot analysis, that the siRNAs silenced their corresponding exon-containing transcripts efficiently (**Fig.** **2C, D**). Both, *TAF4_ex5_siRNA* and *TAF4_ex6_siRNA* resulted in a significant down-regulation of their transcriptional target, *TAF4_v1*, at the mRNA and protein levels as compared to cells transfected with control siRNAs only (**Fig.** **2C, D** and data not shown). As both siRNAs had similar effects on transcriptional silencing of *TAF4_v1,* we further refer to *TAF4_ex5_siRNA* or *TAF4_ex6_siRNA* as *TAF4* siRNA. Intriguingly, silencing of *TAF4_v1* resulted in the up-regulation of alternatively spliced *TAF4_v2* and *TAF4_v5* mRNAs and decreased expression of *TAF4_v4* mRNA levels (**Fig.** **2C**) suggesting a feedback loop in controlling alternative splicing of *TAF4*. Prolonged treatments of hMSCs with *TAF4* siRNAs resulted in the induced expression of *TAF4_v5* mRNAs and significantly reduced expression of *TAF4_v4* mRNAs. However, this alternative splice profile is individual-specific and each donor has its own TAF4 ASVs composition. Western blot analysis data revealed that hMSCs expressed the canonical form of TAF4 protein at relatively low levels, whereas upon RNAi targeting of the hTAF4-TAFH domain the pattern of TAF4 isoforms changed significantly (**Fig.** **2D**). As evidenced in **Figure** **2D** **,** the RNAi induced changes could involve the most abundant isoform TAF4_v2 with the calculated molecular weight of 73 kDa. Given that TAF4 isoforms (TAF4_v1, _v4, _v5, _v6, _v7, _v9) have relatively similar calculated molecular weights, the Western blot resolution was insufficient to make conclusive identification of these isoforms upon RNAi. Accordingly, siRNAs targeting exons V and VI shifted *TAF4* mRNA splicing patterns in human MSCs towards the generation of mRNAs encoding protein isoforms with an altered hTAF4-TAFH domain.

### hTAF4-TAFH controls proliferation and cell cycle exit via TP53 activation and switch from canonical to non-canonical WNT signaling

**Figure 3:**
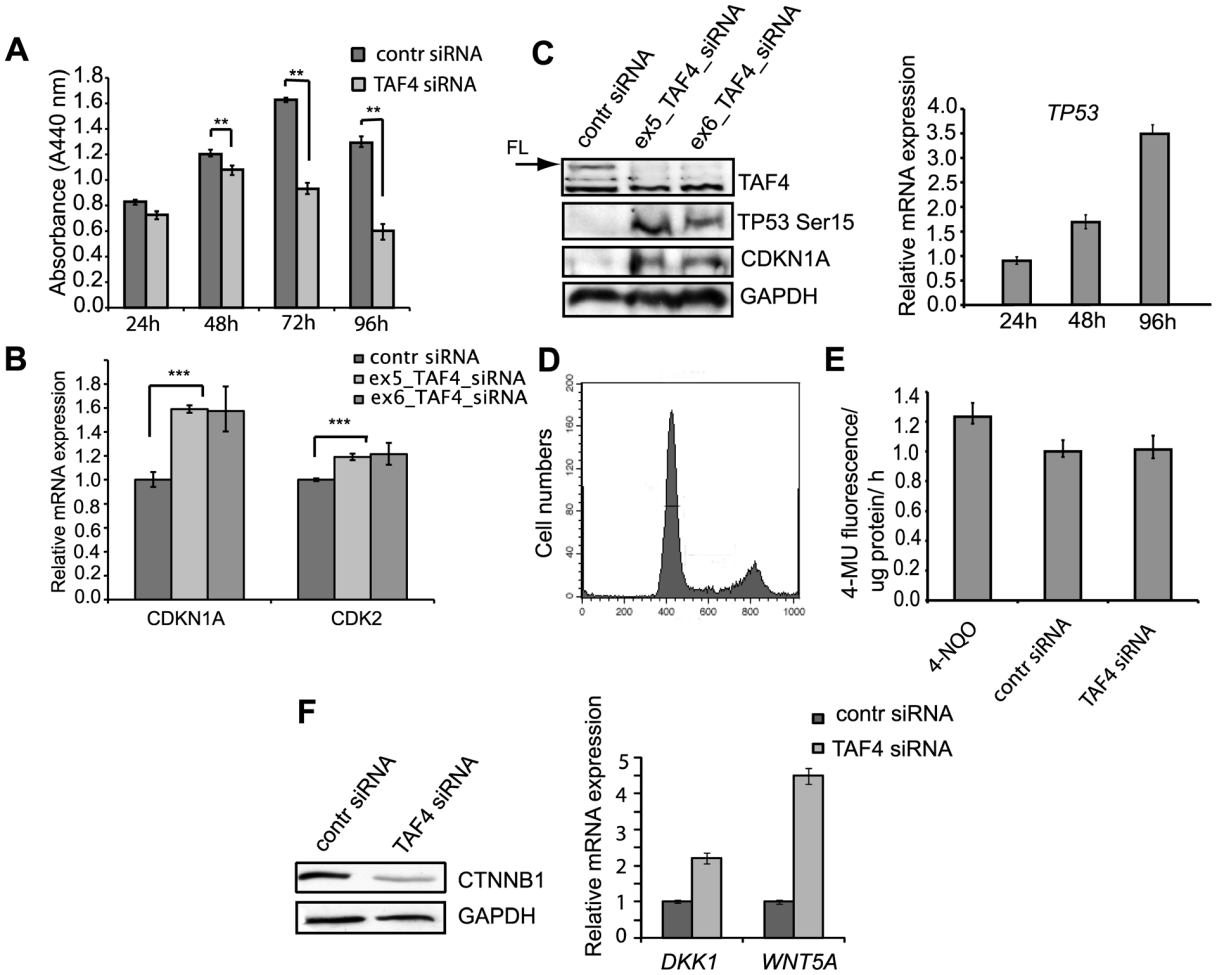
*TAF4* siRNA treatment of human MSCs induces TP53-dependent cell-cycle arrest and switching from canonical to non-canonical WNT signaling. (**A**) Effects of *TAF4* siRNA treatments on hMSC proliferation at different time points as compared to negative control siRNA. hMSCs were transfected with 50 nM of *TAF4* or control siRNAs and analyzed at the indicated time points by WST-1 proliferation assay. Experiments were done in triplicate and a comparison was made to control siRNA treatments (_**_ indicates significant differences between control and *TAF4* siRNA groups with P<0.01). (**B**) Down-regulation of the canonical form of TAF4 affects the expression of cell cycle regulators. hMSCs were transfected with 50 nM *TAF4-*specific (ex5_ or ex6_*TAF4* siRNAs) or control siRNAs. Relative expression of cell cycle regulators *CDKN1A* and *CDK2* as compared to control siRNAs transfected cells was analyzed using real-time RT-PCR at 6 h post-treatment. Observed differences were statistically significant (Student's *t*-test) with *** P<0.001. (**C**) *TAF4* siRNA-mediated RNAi affects expression of cell cycle regulator proteins. Western blot analysis of cell cycle regulators TP53 and CDKN1A/P21 24 h following transfection of hMSCs with control or both, ex5_ and ex6_*TAF4*-specific siRNAs. Expression of TAF4 and GAPDH was analyzed to ensure effective silencing and equal loading *(left)*. Time-dependent expression of *TP53* was analyzed by real-time PCR and compared to the mRNA levels in *TAF4* and control siRNA treated hMSCs *(right)*. (**D**) Down-regulation of the canonical form of TAF4 does not induce apoptosis. FACS analysis of the cell cycle progression of hMSCs treated with *TAF4* siRNAs. (**E**) *TAF4* siRNA treatment doesn't cause senescence of hMSCs. Quantitative SA-ß-gal assay. hMSC extracts were prepared from *TAF4* and control siRNA transfected cells 48 h post-treatment. 1 µM 4-NQO was added to hMSCs for 1 h and used as positive senescence control. Fluorescence intensity of 4-MU hydrolysis was normalized to total protein. Error bars in experiments represent the standard deviations of three independent experiments (P<0.005) (**F**) RNAi of hTAF4-TAFH switches from canonical to non-canonical WNT signaling. 20 µg of control or *TAF4* siRNA-treated whole cell lysates were analyzed by Western blot analysis for the expression of ß-catenin (CTNNB1) and GAPDH as loading control *(left)*. Real-time PCR shows increased expression of non-canonical markers of WNT signaling in *TAF4* siRNA treated hMSCs as compared to control siRNA treated cells. Differences are statistically significant with P<0.001 *(right)*.

Next, we analyzed the effect of the hTAF4-TAFH domain-targeting RNAi on the proliferation and cell cycle of hMSCs (**Fig.** **3**). Silencing of alternative splice variants encoding a structurally intact hTAF4-TAFH domain in hMSCs resulted in growth inhibition as analyzed using WST-1 proliferation assay (**Fig.** **3A**). Already 6 h treatment of hMSCs with *TAF4* siRNAs resulted in the upregulation of *CDKN1A* and *CDK2* levels (**Fig.** **3B**). Western blot analysis revealed that changes in *TAF4_v1* and *CDKN1A* mRNA levels were accompanied by a marked decrease in the expression of the canonical form of TAF4 protein and significant increase of CDKN1A protein levels. Also, we detected accumulation of hyperphoshporylated TP53^Ser15^ in hMSCs following 24 h treatment **(** **Fig.** **3C** **).** At 48 h post-treatment of hMSCs with *TAF4* siRNAs, increased *TP53* transcription was observed (**Fig.** **3C**). These findings demonstrate that depletion of the canonical hTAF4-TAFH activity in hMSC correlates with the cell cycle exit.

Further, we examined whether the observed cell-growth retardation was related to induction of apoptosis. Flow cytometry analysis data clearly showed that the proportion of apoptotic cells in *TAF4* siRNA treated hMSCs was insignificant (**Fig.** **3D**). Propidium iodide staining analysis using the NucleoCounter (data not shown) confirmed these data. To exclude that *TAF4* siRNA triggers TP53-dependent senescence in the human MSCs, quantitative senescence-associated ß-galactosidase assay was performed. Assay results clearly show no increased ß-galactosidase activity in *TAF4* siRNA treated hMSCs compared to control siRNAs at 48 h post-treatments (**Fig.** **3E**). Also, no changes in hMSC morphology were observed following *TAF4* siRNA treatments (data not shown). These results suggest that observed suppression of cell proliferation is associated with cell cycle arrest and not with the induction of cell senescence or apoptosis. Obtained results support the hypothesis of TP53 involvement in TAF4-driven differentiation of hMSC.

Since TP53 activates WNT pathway signaling in mouse embryonic stem cells [39] and the hTAF4-TAFH domain is the direct target of WNT signaling in *Drosophila* [19], we examined the possibility that the WNT pathway is also involved in hTAF4-TAFH governed hMSC proliferation and differentiation. We found that *TAF4* siRNA treatment significantly down-regulated the major player in canonical WNT signaling, ß-catenin, while the expression levels of non-canonical WNT signaling activator *WNT5A* and the inhibitor of the WNT pathway *DKK1* were significantly increased (**Fig.** **3F**). Taken together, these findings provide the first cues that TAF4 protein isoforms with a deleted hTAF4-TAFH domain may function as direct co-activators in the non-canonical WNT signaling pathway that is mediated by JNK, PKC, Ca (2+) or Rho [40].

### hTAF4-TAFH domain integrity supports adipo- and osteogenic and blocks chondrogenic differentiation of hMSCs

**Figure 4:**
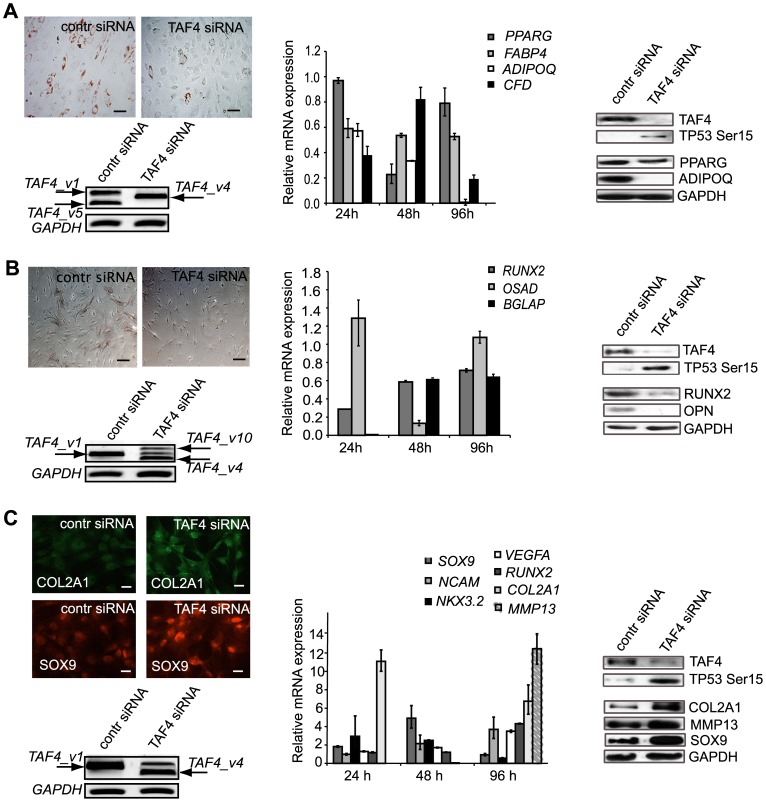
TAFH domain disruption by *TAF4* siRNA attenuates adipogenesis and osteogenesis and accelerates chondrogenesis in human MSCs. (**A**) TAF4 down-regulation slows down adipogenesis in hMSCs. Oil-Red-O staining of lipid droplets of *TAF4* or control siRNA treated and adipogenesis stimulated hMSCs at day 6 post-induction. siRNAs were transfected 24 h prior to the stimulation of differentiation of hMSCs. Scale bar, 40 µm *(top left)*. Western blot analysis reveals reduced expression of adipogenic markers, namely PPARG and ADIPOQ at day 5 after induction of differentiation of *TAF4* siRNA treated hMSCs along adipocyte pathway *(right panel)*. Real-time PCR analysis of the expression of adipocyte markers in hMSCs at different time points of post-transfection and differentiation. The expression is normalized to control siRNA treatments *(middle panel)*. RT-PCR analysis of *TAF4* ASVs expression using *TAF4_v1b* specific primers in *TAF4* or control siRNA transfected and towards adipogenesis stimulated hMSCs at day 5 post-transfection. *GAPDH* mRNA was analyzed for gel loading normalization *(left bottom)*. (**B**) RNAi of hTAF4-TAFH inhibits osteogenic differentiation of hMSCs. Alkaline phosphatase staining of *TAF4* or control siRNA exposed and osteogenesis stimulated hMSCs at day 6 post-induction. siRNAs were transfected 24 h prior to the stimulation of hMSC differentiation. Scale bar, 40 µm *(top left)*. Western blot analysis reveals reduced expression of osteogenic markers RUNX2 and OPN *(right panel)*. Real-time PCR analysis of the expression of osteogenic markers in hMSCs at different time points post-transfection and differentiation. The expression is normalized to negative control siRNA treatment *(middle panel)*. RT-PCR analysis of *TAF4* ASVs expression using *TAF4_v1b* specific primers in *TAF4* or control siRNA treated and stimulated to osteogenic differentiation hMSCs at day 5 post-transfection. *GAPDH* mRNA was analyzed for gel loading normalization *(left bottom)*. (**C**) RNAi of hTAF4-TAFH accelerates chondrogenic differentiation of hMSCs. Immunofluorescence staining analysis of *TAF4* or control siRNA treated hMSCs upon chondrogenic stimulation at day 6 post-treatment reveals induced expression of COL2A1 and SOX9. siRNAs were transfected 24 h prior to stimulation of differentiation of hMSCs. Scale bar, 40 µm *(top left)*. Western blot analysis shows increased expression of chondrogenic marker genes in *TAF4* siRNA treated hMSCs. COL2A1 expression was upregulated at day 2 post-differentiation; SOX9 and MMP13 level of expression were increased at 8 days after siRNA treatments *(right panel)*. Real-time PCR analysis of the expression of chondrogenic markers in hMSCs at different time points post-transfection and differentiation. The expression is normalized to control siRNA treatment *(middle panel)*. RT-PCR analysis of *TAF4* ASV expression using *TAF4_v1b* specific primers in *TAF4* or control siRNA treated hMSCs upon stimulation to chondrogenic differentiation at day 5 post-transfection. *GAPDH* mRNA was analyzed for gel loading normalization *(left bottom)*. Effects of RNAi of hTAF4-TAFH on activation of TP53^Ser15^ were observed in each differentiation study using immunoblot analysis. Real-time PCR differences were found to be statistically significant with P<0.005.

hMSCs were differentiated along adipo-, osteo- and chondrogenic lineages upon treatments with *TAF4* siRNAs or control siRNAs following analysis of expression of appropriate lineage-specific markers using Western blot and quantitative RT-PCR to analyze the effects of hTAF4-TAFH on the differentiation potential of hMSCs. Effective downregulation of the canonical form of TAF4 protein along with the hyperphoshporylation of TP53^Ser15^ was observed in siRNA treated cells **(** **Fig** **4** **A–C)**. In addition, RNAi treatments followed by differentiation resulted in the induction of expression of *TAF4* splice variants encoding protein isoforms with a modified hTAF4-TAFH domain (**Fig** **4** **A–C**).

On day 6 of adipogenic differentiation, specific Oil-Red-O staining revealed intensely reactive lipid droplets in control vehicle-treated cells **(** **Fig.** **4A** **)**, indicating adipogenic differentiation, whereas far less intensive staining was observed in *TAF4* siRNA transfected hMSCs. Analysis of expression of adipose-specific PPARG and ADIPOQ at protein and RNA levels indicated a significantly delayed adipogenic differentiation of hMSCs exposed to *TAF4* siRNAs targeting hTAF4-TAFH **(** **Fig.** **4A** **)**. These findings show that RNAi silencing of hTAF4-TAFH activity blocks adipogenesis in hMSCs.

To assess the effect of hTAF4 RNAi on osteogenic differentiation, hMSCs were treated with *TAF4* or control siRNAs and cultured in osteogenic differentiation medium for 5 days. Alkaline phosphatase (AP) staining confirmed effective osteogenic differentiation in control siRNA-treated cells, whereas much less AP + cells were observed in *TAF4* siRNA treated hMSCs upon differentiation **(** **Fig.** **4B** **)**. Also, immunoblot analysis using anti-RUNX2 and anti-OPN antibodies showed decreased levels of expression of these osteogenic markers in *TAF4* siRNA transfected hMSCs upon differentiation **(** **Fig.** **4B** **)**. Surprisingly, the expression of *RUNX2* and *BGLAP* mRNAs that initially (24 h post-treatment) decreased significantly, reached the control level by day 5 of differentiation. Substantially decreased levels of *OSAD* expression were detectable only 48 h post-differentiation **(** **Fig.** **4B** **)**. All the data evidenced severely delayed osteogenesis in hMSC cells with low hTAF4-TAFH activity.

Next, we examined the role of hTAF4-TAFH in chondrogenic differentiation. Immunofluorescence analysis of *TAF4* and control siRNA-treated hMSCs that were subjected to chondrogenic differentiation for 6 days showed intense staining with anti-COL2A1 and anti-SOX9 antibodies in TAF4-depleted hMSCs, indicating enhanced chondrogenic differentiation in these cells **(** **Fig.** **4C** **)**. In close agreement with these results, immunoblot analysis data further confirmed the up-regulation of COL2A1 protein expression by day 1, SOX9 expression by day 2 and MMP13 expression by day 5 **(** **Fig.** **4C** **)**, supporting the evidence of accelerated chondrogenesis. To analyze the expression of chondrogenic markers upon *TAF4* siRNA treatments, a real-time RT-PCR analysis was performed using a set of gene-specific primers. Increased levels of *SOX9* mRNA, with a peak at day 2, rapid upregulation of *NKX3.2* and *COL2A1* mRNAs by day 1, gradually rising levels of expression of *NCAM, VEFGA, RUNX2* mRNAs and the appearance of *MMP13* mRNA later in the differentiation process were observed in *TAF4* siRNA treated cells compared with that of the control vehicle treated hMSCs upon chondrogenic differentiation **(** **Fig.** **4C** **)**. Thus, based on the immunofluorescence and gene expression analysis data it was concluded that the activity of the canonical hTAF4-TAFH suppresses chondrogenesis.

Notably, the levels of *TAF4_v1* mRNA decreased substantially whereas those of *TAF4* mRNAs encoding protein isoforms with a modified hTAF4-TAFH domain (*TAF4_v2*, *TAF4_v4, TAF4_v10*, and other rare transcripts (data not shown)) accumulated upon *TAF4* RNAi treatments and during the further course of differentiation of hMSCs **(** **Fig.** **4A–C** **)**. The data is in close agreement with data on the expression of *TAF4* splice variants in the differentiated hMSCs **(** **Fig.** **2B** **)**, which altogether strongly support the herein described findings that hTAF4-TAFH domain integrity is required for adipo- and osteogenic differentiation and controls chondrogenic differentiation.

## Discussion

Here, we present the first description of alternatively spliced *TAF4* mRNAs carrying deletions of exons encoding the hTAF4-TAFH domain. We describe the functional consequences of the structural integrity of the hTAF4-TAFH domain on cellular differentiation. Our data reveals that differentiation of hMSCs along adipo-, osteo- and chondrogenic lineages is, at least in part, regulated by hTAF4-TAFH domain activity, with possible cross-talks to the early activity of TP53 and switching of WNT signaling from a canonical to a non-canonical pathway.

### Variety of hTAF4 AS variants

Our data, along with published findings [30], shows a broad distribution of alternative splice variants across cells and tissues of *TAF4* alternative mRNAs. Here, we show that across the *TAF4* gene, alternative splicing predominantly targets exons VI and VII. These exons encode the hTAF4-TAFH domain, a five-helix structure that is responsible for protein-protein interactions and recognizes a hydrophobic DΨΨζζΨΦ motif (similar to LxxLL) of TAF4 interaction partners [18]. Alternative splicing of exons VI and VII alters the flat and wide binding surface in the hTAF4-TAFH domain, making it more similar to that of the ETO-TAFH domain. ETO-TAFH-dependent interactions with LxxLL-carrying proteins, including LZIP, E-proteins, nuclear hormone receptors and subunits of Mediator complex have been suggested to affect the whole PIC composition and activity [41]. Thus, splicing events in the exons coding the hTAF4-TAFH domain ultimately contribute to the changes in target specificity, perhaps allowing the fine-tuning of a transcriptional response to activators that are important during development. Close support for this assumption stems from our data showing that in response to *TAF4* RNAi treatments, hMSCs start to express alternative splice variants of *TAF4*, namely *TAF4_v2, TAF4_v4* or *TAF4_v5* mRNAs that encode protein isoforms with only 2 helixes out of five preserved from the canonical hTAF4-TAFH structure. It is highly likely that changed co-activator properties of the hTAF4-TAFH domain may influence overall TFIID complex stability, PIC assembly and the basal transcription, having functional consequences in the selection of developmental pathways in human cells.

It requires further clarification, whether *TAF4* alternative splice variants with ORF preservation will encode functional proteins or could act as lncRNAs. In both cases, *TAF4* ASVs impact the ability of TAF4 to promote the cell cycle and control differentiation. ASVs retaining canonical ORF are co-expressed although at different levels than *TAF4* ASVs encoding protein isoforms with altered hTAF4-TAFH activity. For example, the expression levels of *TAF4_v1* mRNA encoding the canonical form of the protein with intact hTAF4-TAFH are comparable with the sum of other ASVs in differentiated hMSCs. Therefore, it is likely that the relative levels of *TAF4* ASVs and mutual cross-talk (cooperation or competition) will influence the final biological outcome. The expression of some *TAF4* ASVs is ubiquitous while of the others are tissue-specific (our data). Differences in the levels of expression of *TAF4* ASVs were observed in all individuals analyzed (**Fig.** **2A**) and distinguished by every hMSC donor [42]. In addition, differences in silencing or activation of various *TAF4* ASVs were also observed throughout the study (**Fig.** **2A** **,** **4**). These individual-specific diversity could be related to the differences in the chromatin and epigenetic background as it has been shown that siRNAs that target exonic sequences in the close proximity to alternatively spliced exons could regulate splicing in a chromatin and epigenetic context-dependent manner [43].

Therefore apparently, the numerous *TAF4* ASVs with altered hTAF4-TAFH activity may permit a differential regulation of TAF4 functions during cell differentiation. Yet, this hypothesis awaits further clarification.

### Molecular mechanisms of hTAF4-TAFH-activity-mediated differentiation

hMSCs that were differentiated into adipo-, osteo- or chondrocytes showed complex expression patterns of *TAF4* ASVs encoding proteins with altered hTAF4-TAFH. Recently, using modified versions of TAF4 protein it was shown that ETO-TAFH domain of TAF4 is targeted by WNT signaling in *Drosophila* [44], [19]. In addition to global gene silencing that has been evidenced to occur during ES cell differentiation [45], proteasome-dependent TAF4 degradation was observed in F9 embryonic carcinoma cells in response to retinoic acid-induced differentiation [46]. As these processes are related to cell fate determination and control of stem cell proliferation, this prompted us to investigate the consequences of hTAF4-TAFH inactivation on hMSCs proliferation and differentiation.

RNAi targeting of exons V and VI of *TAF4* resulted in cell cycle arrest and accumulation of hyperphosphorylated TP53^Ser15^ protein in hMSCs. TP53 activation via phosphorylation is associated with the induction of apoptotic cell death or irreversible cell-cycle exit, commonly termed cellular senescence [47]. Both of these processes are linked to cellular differentiation. Depending on the state and cellular environment, TP53 exerts a regulatory role on various differentiation programs [48]–[54]. Induction of TP53 expression represses cell proliferation and through *miR-34a* and *miR-145* activation downregulates pluripotent stem cell factors, such as OCT4, KLF4, LIN28A, and SOX2 thereby affecting differentiation and human cell state [55]. Our data on the interrelations of TP53 and TAF4 are in close agreement with published data related to TFIID complex subunits. The detailed mechanism of TAF4-TP53 interaction remains to be established, but our data allows for suggesting the involvement of TP53 in TAF4-dependent differentiation of hMSCs. Indeed, TAF4 was detected on a TP53-binding site on a *CDKN1A/P21* promoter and its binding to the promoter increased in response to UV irradiation [56]. TAF9 has been shown to regulate the stability and activity of TP53 by binding to its N-terminally located transcription activation domain [57], [58]. Functional interactions between TP53 and TAF3 [59], TAF1 [60], TAF6 [61] and TBP [62] have been reported. Altogether, the data suggests that while TAF4 may be involved in the stimulation of cell differentiation by alternative splicing generating protein isoforms with varying hTAF4-TAFH, the down-stream activation of p53 pathways executing this differentiation process are likely to play an important role.

WNT signaling promotes activation of WNT target genes by targeting Pygopus-TAFH interactions in *Drosophila* [44]. Active canonical WNT signaling stimulates osteogenesis in certain cellular contexts [63], [64]. However, interplay between canonical ß-catenin-dependent and a variety of non-canonical pathways has been evidenced to guide cells to differentiate along defined pathways and also directs cell fate decisions of hMSCs [40]. Several members of the WNT family have been shown to block osteogenesis and adipogenesis. For example, a non-canonical ligand WNT5A inhibits the ability of PPARG to activate its target genes and ultimately thus prevents adipogenesis [65]. The positive impact of WNT inhibitor DKK1 on early chondrogenesis has also previously been documented [66]. Furthermore, to confirm the interplay of TP53 and WNT signaling that was observed by us in *TAF4* siRNA treated cells, previous studies have shown that TP53 and its target *miR-34* suppress canonical WNT signaling [67]. Our data showing reduced levels of ß-catenin in response to decreased expression of *TAF4_v1* with hTAF4-TAFH intact are in close agreement with the findings described above. Taken together, we suggest that abrogation of hTAF4-TAFH activity by expression of TAF4 protein isoforms with an hTAF4-TAFH altered structure has severe consequences on the co-activator function of TAF4 in canonical WNT signaling. In order to counter-balance the inhibition of the canonical WNT pathway, compensation by activation of the non-canonical pathway by WNT5A takes place and acts as a mediator of the induction of chondrogenesis in hMSCs upon differentiation. Similar findings have been described by Bradley et al [68].

RNAi targeting of hTAF4-TAFH activity results in down-regulation of *TAF4_v1* and promotes chondrogenesis by inhibiting or delaying osteo- and adipogenesis of human adipose derived hMSCs. Differentiation along osteo- and adipogenic lineages verified by using expression analysis of appropriate markers was delayed but not completely inhibited upon RNAi targeting of hTAF4-TAFH activity. Since close interplay between osteogenesis and chondrogenesis is regulating the early development of bone, and RUNX2 modulates both of these differentiation programs, it is also possible that downregulation of hTAF4-TAFH activity in hMSCs influences *RUNX2* turnover in osteogenesis. Also, TP53 deficiency has been shown to enhance osteogenesis via SMAD1 signaling in mice [69], thereby providing a strong possibility that low levels of hTAF4-TAFH activity promote a chondrogenic switch in mesenchymal cells with activated TP53.

### Depletion of hTAF4-TAFH is necessary for normal development

Inactivation of individual TAFs in *Drosophila* and mammalian cells have demonstrated that TAFs are not essential for the transcription of all RNA pol II-dependent genes and in fact, there is a great variation in target genes of individual TAFs [28], [70]–[72]. For example, myoblasts shed most of the subunits of TFIID complex, apart from TAF3 and a TBP homolog TRF3, in the process of differentiation to myotubes [73], [74]. Expression of several TAF subunits, with the exception of TAF8, was observed to be downregulated upon differentiation of 3T3-L1 preadipocytes into adipocytes [75]. Inactivation of TAF10 affects liver development and stability of the TFIID complex as a whole [76]. Furthermore, TAF7 has recently been shown to be necessary for mouse embryonic development but not for the survival and differentiation of mature T cells [21]. In *Drosophila*, targeting of TAF4 activity by RNAi yields in the reduced levels of TBP, TAF6, and TAF9 along with a severe loss in TAF1 and TAF5 protein expression without affecting their mRNA levels [77]. Reduction of TAF4 activity has been shown to have the most dramatic effects on transcription as compared with other subunits of TFIID in *C.elegans* [23]. All the data, along with our findings on TAF4 function, are consistent with the suggestion that regulated degradation of defined TFIID subunits or controlling their activity via alternative splicing, as in case of TAF4 demonstrated here, is required for directing the normal cellular differentiation process.

In conclusion, altered splicing and regulated expression of *TAF4* alternative mRNAs encoding protein isoforms with altered hTAF4-TAFH activity govern the cell-cycle progression in hMSCs through expression of cell cycle inhibitors and TP53 activation that support realization of specific differentiation programs. Our data also reveals the potential role of TAF4 isoforms in delaying adipogenic differentiation of hMSCs and thus contributes to the understanding of the mechanisms of obesity. In adipose-derived hTAF4-TAFH-depleted hMSCs, chondrogenesis is the most preferable differentiation program. Cellular mechanisms leading to such transitions are currently not clear and should be clarified with future studies, but our research suggests the role of TP53 along with the switching of WNT signaling from a canonical to a non-canonical pathway in response to predominant expression of TAF4 ASVs with abrogated hTAF4-TAFH activity in human mesenchymal stem cells.

## Supporting Information

Table S1 Description of hMSCs clones used in the study.
(DOCX)

Table S2 List of primers used in the study.
(DOCX)
