# Supplementary material for: Alternative Splicing Targeting the hTAF4-TAFH Domain of TAF4 Represses Proliferation and Accelerates Chondrogenic Differentiation of Human Mesenchymal Stem Cells
Source: PLoS One. 2013 Oct 2;8(10):e74799. doi: 10.1371/journal.pone.0074799 (PMC3788782; doi:10.1371/journal.pone.0074799)
Supplement: Table S1 — Description of hMSCs clones used in the study. (DOCX) [file pone.0074799.s001.docx]

| **Clone** | **Sex** | **Age** | **Part of body** | **Used in the illustrations of the study** |
| --- | --- | --- | --- | --- |
| hMSC_1 | ND | ND | lipoaspirate | isoform-specific RT-PCR (Fig. 2A); proliferation and cell cycle (Fig. 3A-E) |
| hMSC_2 | F | 83 | scar adipose tissue from abdomen | isoform-specific RT-PCR (Fig. 2A), differentiation and isoform-specific RT-PCR (Fig. 2B) |
| hMSC_3 | F | 27 | adipose tissue from abdomen | isoform-specific RT-PCR (Fig. 2A); proliferation and cell cycle (Fig. 3A-E); *TAF4* siRNA treatment and differentiation (Fig. 4) |
| hMCS_4 | F | 48 | adipose tissue from abdomen | isoform-specific RT-PCR (Fig. 2A), time-course siRNA treatment (Fig. 2C), *TAF4* siRNA treatment Western blot (Fig. 2D); WNT pathway markers (Fig. 3F) |
| hMSC_5 | F | 53 | adipose tissue from abdomen | isoform-specific RT-PCR (Fig. 2A); proliferation and cell cycle (Fig. 3A-E); |
| hMSC_6 | M | 71 | adipose tissue from crural area | WNT pathway markers (Fig. 3F); *TAF4* siRNA treatment and differentiation (Fig. 4) |
| hMSC_7 | F | 41 | adipose tissue from abdomen | proliferation and cell cycle (Fig. 3A-E); WNT pathway markers (Fig. 3F); *TAF4* siRNA treatment and differentiation (Fig. 4) |
