# Supplementary material for: Alternative Splicing Targeting the hTAF4-TAFH Domain of TAF4 Represses Proliferation and Accelerates Chondrogenic Differentiation of Human Mesenchymal Stem Cells
Source: PLoS One. 2013 Oct 2;8(10):e74799. doi: 10.1371/journal.pone.0074799 (PMC3788782; doi:10.1371/journal.pone.0074799)
Supplement: Table S2 — List of primers used in the study. (DOCX) [file pone.0074799.s002.docx]

| Gene | Sense primer | Antisense primer |
| --- | --- | --- |
| *TAF4_v1a* | CTGCCACGGAAACTATGGAA | CTTTACAGGACCGCTTAGC |
| *TAF4_v1b* | CTGCCACGGAAACTATGGAA | GTCTGCGTCAACGTCACCT |
| *TAF4_v4* | CTCGTGCAGAATTTACTGGTCA | GCCTGCTCATATCTGTCGTCAT |
| *TAF4_v5* | CTTGTGCCTTTCCTGAAGGTC | CTTTACAGGACCGCGTTAGC |
| *TAF4_v2* | TCGTGCAGAATTTACTGGGAC | GCCTGCTCATATCTGTCGTCAT |
| *CDK2* | CCTCTGCTCTCACTGGCATT | GGACTCCAAAAGCTCTGGCTA |
| *CDKN1A* | CACTGTCTTGTACCCTTGTGC | GGATTAGGGCTTCCTCTTGG |
| *TP53* | TGGAGGAGCCGCAGTCAGATCC | TTGCTTGGGACGGCAAGGGG |
| *DKK1* | ATAGCACCTTGGATGGGTATTCC | CACAGTCTGATGACCGGAGA |
| *WNT5A* | ATGGCTGGAAGTGCAATGTCT | ATACCTAGCGACCACCAAGAA |
| *PPARG2* | CCTATTGACCCAGAAAGCGATT | CATTACGGAGAGATCCACGGA |
| *FABP4* | GGGTCACAGCACCCTCCTGAAAAC | GGTTTGGCCATGCCAGCCACT |
| *ADIPOQ* | TGCACAGGTTGGATGGCGGG | GGGACCTTCAGCCCCGGGTA |
| *CFD* | CACCGAGCGCTTGATGTGCG | TCCCGGGCTTCTTGCGGTTG |
| *RUNX2* | GTGGTCCTATGACCAGTCTTAC | CTCACGTCGCTCATCTTGC |
| *OSAD* | GTAGAACTCAGTGTTGGACACAAC | GGTCAATAGAAGGACACATCACTG |
| *BGLAP* | CGCTACCTGTATCAATGGCTGG | CTCCTGAAAGCCGATGTGGTCA |
| *SOX9* | CAGACGCACATCTCCCCCAAC | CTCTCGCTTCAGGTCAGCCTTG |
| *NCAM* | GAACGGAGGAGGAGAGGACC | CCTTTGTCTGTGTGGCGTCA |
| *NKX3.2* | CCGCTTCCAAAGACCTAGAGGA | ACCGTCGTCCTCGGTCCTTGG |
| *VEGFA* | CCTGGTGGACATCTTCCAGGAGTACC | GAAGCTCATCTCTCCTATGTGCTGGC |
| *COL2A1* | CTGGTATTGCTGGCTTCAAAGG | AGACCATCTTGACCTGGGAAA |
| *MMP13* | CAGGAAACCAGGTCTGGAGAT | CAAACTGTATGGGTCCGTTG |
